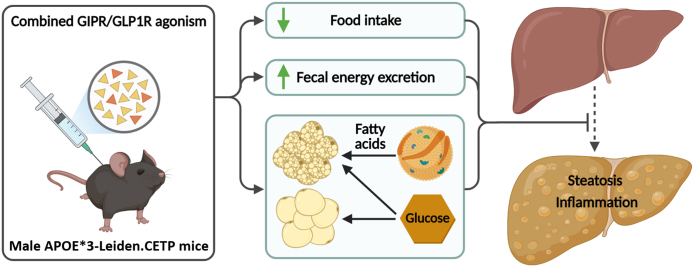# Combined GIP receptor and GLP1 receptor agonism attenuates NAFLD in male APOE∗3-Leiden.CETP mice

**DOI:** 10.1016/j.ebiom.2023.104684

**Published:** 2023-06-26

**Authors:** Zhixiong Ying, Robin van Eenige, Xiaoke Ge, Christy van Marwijk, Joost M. Lambooij, Bruno Guigas, Martin Giera, Jan Freark de Boer, Tamer Coskun, Hongchang Qu, Yanan Wang, Mariëtte R. Boon, Patrick C.N. Rensen, Sander Kooijman

**Affiliations:** a Division of Endocrinology, Department of Medicine, Leiden University Medical Center, Leiden, the Netherlands; b Einthoven Laboratory for Experimental Vascular Medicine, Leiden University Medical Center, Leiden, the Netherlands; c Department of Parasitology, Leiden University Medical Center, Leiden, the Netherlands; d Department of Cell and Chemical Biology, Leiden University Medical Center, Leiden, the Netherlands; e The Center for Proteomics and Metabolomics, Leiden University Medical Center, Leiden, the Netherlands; f Departments of Pediatrics and Laboratory Medicine, University of Groningen, University Medical Center Groningen, Groningen, the Netherlands; g Department of Diabetes/Endocrine, Lilly Research Laboratories, Lilly Corporate Center, Indianapolis, IN, United States; h Med-X Institute, Center for Immunological and Metabolic Diseases and Department of Endocrinology, First Affiliated Hospital of Xi'an Jiaotong University, Xi'an Jiaotong University, Xi'an, China

**Keywords:** Adipose tissue, Bile acid metabolism, Hepatic steatosis, Hepatic inflammation, Glucose metabolism, Lipid metabolism

## Abstract

**Background:**

Combined glucose-dependent insulinotropic polypeptide receptor (GIPR) and glucagon-like peptide-1 receptor (GLP1R) agonism is superior to single GLP1R agonism with respect to glycemic control and weight loss in obese patients with or without type 2 diabetes. As insulin resistance and obesity are strong risk factors for nonalcoholic fatty liver disease (NAFLD), in the current study we investigated the effects of combined GIPR/GLP1R agonism on NAFLD development.

**Methods:**

Male APOE∗3-Leiden.CETP mice, a humanized model for diabetic dyslipidemia and NAFLD when fed a high-fat high-cholesterol diet, received subcutaneous injections with either vehicle, a GIPR agonist, a GLP1R agonist, or both agonists combined every other day.

**Findings:**

GIPR and GLP1R agonism reduced body weight and additively lowered fasting plasma levels of glucose, triglycerides and total cholesterol. Strikingly, we report an additive reduction in hepatic steatosis as evidenced by lower hepatic lipid content and NAFLD scores. Underlying the lipid-lowering effects were a reduced food intake and intestinal lipid absorption and an increased uptake of glucose and triglyceride-derived fatty acids by energy-combusting brown adipose tissue. Combined GIPR/GLP1R agonism also attenuated hepatic inflammation as evidenced by a decreased number of monocyte-derived Kupffer cells and a reduced expression of inflammatory markers. Together, the reduced hepatic steatosis and inflammation coincided with lowered markers of liver injury.

**Interpretation:**

We interpretate that GIPR and GLP1R agonism additively attenuate hepatic steatosis, lower hepatic inflammation, ameliorate liver injury, together preventing NAFLD development in humanized APOE∗3-Leiden.CETP mice. We anticipate that combined GIPR/GLP1R agonism is a promising strategy to attenuate NAFLD progression in humans.

**Funding:**

This work was supported by a grant from the Netherlands CardioVascular Research Initiative: the Dutch Heart Foundation, Dutch Federation of University Medical Centers, the 10.13039/501100001826Netherlands Organization for Health Research and Development, and the Royal Netherlands Academy of Sciences [CVON-GENIUS-II] to P.C.N.R., a Lilly Research Award Program [LRAP] Award to P.C.N.R. and S.K., a Dutch Heart Foundation [2017T016] grant to S.K., and an NWO-VENI grant [09150161910073] to M.R.B.; J.F.D.B. is supported by the Nutrition and Health initiative of the University of Groningen; Z.Y. is supported by a full-time PhD scholarship from the 10.13039/501100004543China Scholarship Council (201806850094 to Z.Y.).

**Research in context**

**Evidence before this study**

Combining glucose-dependent insulinotropic polypeptide receptor (GIPR) with glucagon-like peptide-1 receptor (GLP1R) agonism was found to be superior to single GLP1R agonism with respect to glycemic control and weight loss in obese patients with or without type 2 diabetes. Recent mechanistic studies suggest a role of both GIPR and GLP1R signaling in (postprandial) lipid handling and controlling inflammation, beyond their well-known effects on food intake and incretin action.

**Added value of this study**

We show an additive reduction of hepatic steatosis, hepatic inflammation and liver injury upon combined GIPR/GLP1R agonism in high-fat high-cholesterol diet-fed male APOE∗3-Leiden.CETP mice, a humanized model for nonalcoholic fatty liver disease (NAFLD). Mechanistically we report reduced food intake, reduced intestinal lipid absorption, and increased lipid uptake by adipose tissue.

**Implications of all the available evidence**

We demonstrate that combined GIPR/GLP1R agonism attenuates the development of NAFLD in APOE∗3-Leiden.CETP mice. Given the translational value of this model, we anticipate that combined GIPR/GLP1R agonism is a promising strategy to attenuate NAFLD progression in humans as well.

## Introduction

Nonalcoholic fatty liver disease (NAFLD) refers to a spectrum of liver abnormalities ranging from simple hepatic steatosis to nonalcoholic steatohepatitis (NASH). Obesity, diabetes and dyslipidemia are strong risk factors,^1^^,^^2^ and as such NAFLD has become a major health problem affecting an estimated 30% of the global population.^3^ Although several candidates are in the pipeline for the treatment of NAFLD/NASH,^4^^,^^5^ no medication has been approved yet.

Glucagon-like peptide-1 (GLP1), secreted by enteroendocrine L cells in response to nutrient ingestion, potentiates glucose-stimulated insulin release from pancreatic β-cells. For this reason, GLP1 receptor (GLP1R) agonists are widely used in patients with type 2 diabetes.^6^ In addition, GLP1R agonists have been approved for the treatment of obesity due to their suppressive effects on appetite and food intake.^7^ The combined effect of GLP1R agonism on glycemic and body weight control makes it an attractive pharmacological strategy for NAFLD. Indeed, GLP1R agonism has been shown to reduce^8^ or even reverse^9^^,^^10^ hepatic steatosis in mice. Most strikingly, the GLP1R agonists liraglutide (NCT01237119)^11^ and semaglutide (NCT02970942)^12^ also appear to promote the resolution of NASH in humans.

The proposed complementary actions of glucose-dependent insulinotropic polypeptide (GIP), secreted by enteroendocrine K cells in response to food intake, on glycemic control and lowering body weight,^13^^,^^14^ led to the development of dual GIP receptor (GIPR) and GLP1R agonists. Indeed, in diet-induced obese mice, GIPR/GLP1R agonism showed superior efficacy when compared with selective GLP1R agonism.^15^ Also in patients with obesity with or without type 2 diabetes, treatment with the GIPR and GLP1R dual agonists NNC0090-2746 (RG7697)^16^ or tirzepatide (LY3298176)^17^^,^^18^ showed superior glycemic control and weight loss when compared to selective GLP1R agonism.

Recent insights in the mode of action of both GIPR and GLP1R agonism suggest involvement in (postprandial) lipid handling and inflammation as well,^6^^,^^13^^,^^19^ which may also impact NAFLD development. Nonetheless, the effect of combining GIPR and GLP1R agonism in the treatment of NAFLD is still unknown. In the current study, we therefore investigated the effects of GIPR/GLP1R agonism on NAFLD development in male APOE∗3-Leiden.CETP (E3L.CETP) mice, a translational humanized mouse model for exploring the effects of pharmacological interventions on insulin resistance, diabetic dyslipidemia and NAFLD.^20^ We demonstrate that GIPR and GLP1R agonism additively attenuate hepatic steatosis, lower inflammation, ameliorate liver injury, and together prevent the development of NAFLD.

## Methods

### Animals and treatments

**Fig. 1:**
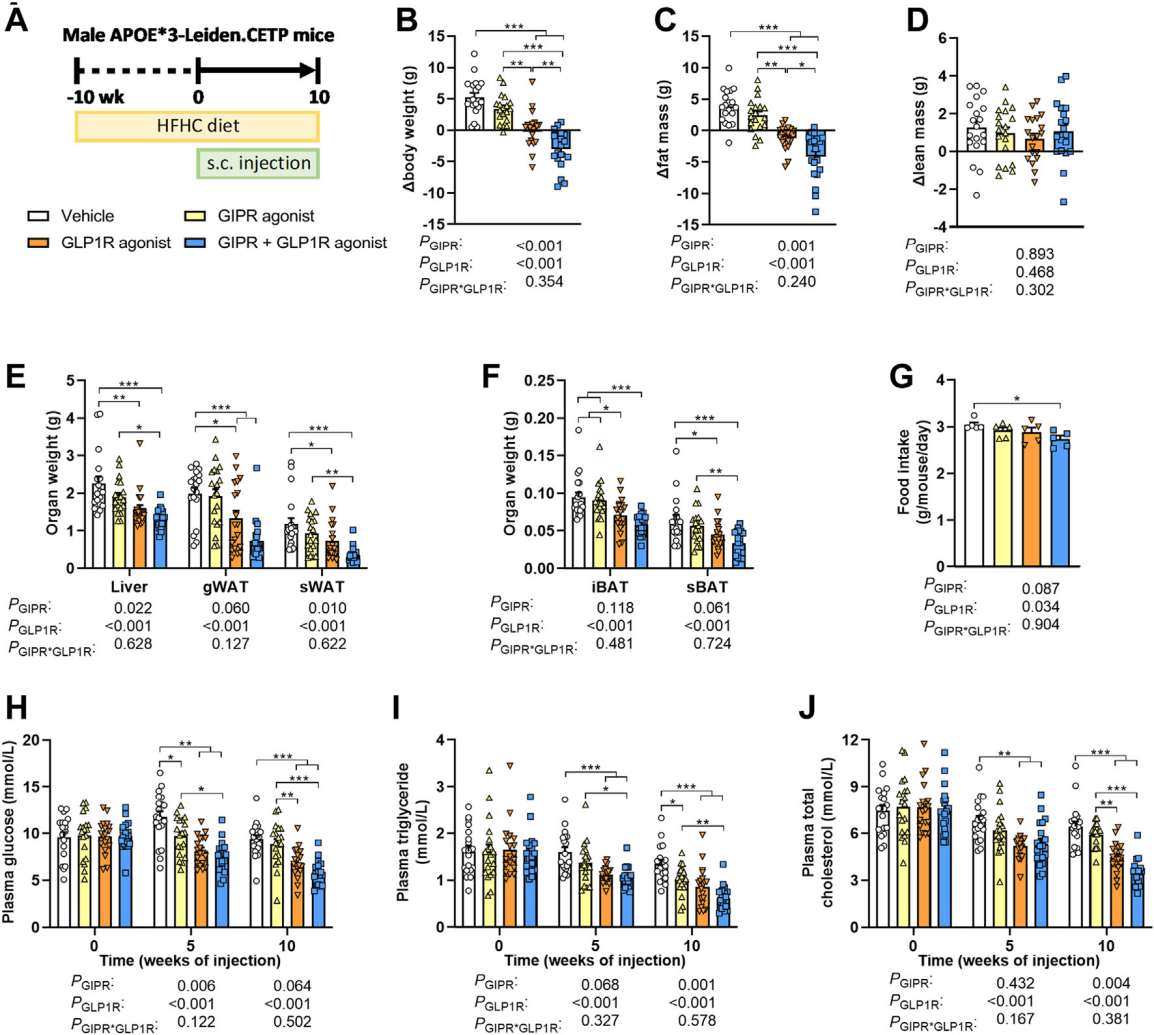
**Combined GIPR/GLP1R agonism lowers fat mass and food intake, which is accompanied by reduced plasma glucose and lipid levels. (A)** Male APOE∗3-Leiden.CETP mice were fed a high-fat high-cholesterol (HFHC) diet and received subcutaneous (s.c.) injections with either a GIPR agonist (GIPFA-085; 300 nmol/kg), a GLP1R agonist (GLP-140; 30 nmol/kg), both agonists at these doses, or vehicle every other day. **(B)** Body weight, **(C)** fat mass and **(D)** lean mass were determined before and after the intervention period and expressed as change from baseline. After 10 weeks of treatment, the weight of **(E)** the liver, gonadal and subcutaneous white adipose tissue (gWAT; sWAT) and **(F)** interscapular and subscapular brown adipose tissue (iBAT; sBAT) were determined. **(G)** Average food intake throughout the intervention period was determined. In 4-h fasted plasma collected at baseline and after 5 and 10 weeks of treatment, the levels of **(H)** glucose, **(I)** triglyceride and **(J)** total cholesterol were measured. Data are presented as mean ± SEM and individual data points. **A**–**F**, **H**–**J***n* = 17–19 mice; **G** derived from *n* = 5 cages per group. *P* values of two-way ANOVA are depicted below figure panels and symbols reflect statistical differences between groups as determined by Tukey post-hoc analysis with *∗P* < 0.05, *∗∗P* < 0.01 and *∗∗∗P* < 0.001.

Hemizygous APOE∗3-Leiden (E3L) mice were crossbred with homozygous human cholesteryl ester transfer protein (CETP) transgenic mice to generate E3L.CETP (C57Bl/6J background) mice as described before.^21^ Male E3L.CETP mice (8–15 weeks of age) were housed under standard conditions (*i.e.,* group housing, 12 h:12 h light–dark cycle, room temperature of 22 °C) and had *ad libitum* access to water and a high-fat high-cholesterol (HFHC) diet (60 KJ% fat + 1% w.w^−1^ cholesterol, Ssniff Spezialdiäten GmbH). After a 10-week run-in period, mice that responded well to the diet^22^ were divided into four treatment groups (*n* = 19 mice per group; in total 76 mice), which were balanced for age, body weight, body composition and plasma levels of glucose, triglycerides (TGs) and total cholesterol (TC) using RandoMice version 1.0.9.^23^ Sample size was determined to be able to show a difference of 25% in hepatic lipid area using ANOVA, assuming a standard deviation of 22%, α = 5%, β = 80% and six pairwise comparisons. Mice were subcutaneously injected every other day with either a GIPR agonist (GIPFA-085; 300 nmol kg^−1^), a GLP1R agonist (GLP-140; 30 nmol kg^−1^), both agonists at the indicated doses, or vehicle (0.02% Tween-80/20 mM Tris/HCl at pH 8.0) for 10 weeks while they were maintained on the HFHC diet (Fig. 1A). Body weight and body composition were measured directly before and at the end of the 10-week intervention period, and food intake was determined weekly by weighing the food in the cage. 4-hour fasted tail vein blood was drawn before the intervention and after 5 and 10 weeks of the intervention.

In a predefined subgroup of mice (*n* = 9 per group, randomly selected as described above; cohort 1), oral glucose tolerance was determined after 5 weeks of treatment. At the end of the 10-week intervention period, organ distribution of intravenously injected glucose and TG-derived fatty acids (FAs) was determined as detailed below, and additional pieces of livers were collected for further analyses.

In the remainder of mice (*n* = 10 per group; cohort 2), oral lipid tolerance was determined after 5 weeks of treatment. The same set of mice were single-housed for 48 h in week 8 of the intervention to collect feces samples. At the end of the 10-week intervention period, mice were killed via CO_2_ inhalation, perfused with PBS, and livers were collected for isolation of hepatic leukocytes (see below) and for further analyses.

### Body weight and body composition

Mice were weighed using a regular weighing scale, and body composition was determined using an EchoMRI-100 (EchoMRI).

### Plasma glucose and lipid levels

Plasma obtained from tail vein blood was used to determine plasma levels of glucose (10786, Human, Germany), TGs (10166588130, Roche Diagnostics) and TC (11489232216, Roche Diagnostics) using enzymatic kits.

### Oral glucose and lipid tolerance test

Directly after the collection of 4-h fasted blood after 5 weeks of intervention, mice received an oral administration of either d-glucose (2 g kg^−1^ in approx. 200 μL water) to assess oral glucose tolerance (cohort 1) or olive oil (7.5 μL kg^−1^) to assess lipid tolerance (cohort 2). For the oral glucose tolerance test (OGTT), plasma was collected from tail vein blood 5, 10, 15, 30, 60 and 120 min later to assess glucose levels as described above. Plasma collected just prior to the oral glucose administration was additionally used to determine insulin levels using Ultra Sensitive Mouse Insulin ELISA Kit (Crystal Chem), which, together with the glucose level, was used to calculate the homeostatic model assessment for insulin resistance (HOMA-IR) using the formula: glucose level (mM) · insulin level (μU/mL) · 22.5^−1^. For the oral lipid tolerance test (OLTT), plasma was collected from tail vein blood 1, 2, 4 and 6 h after the oral lipid bolus to assess TG levels as described above.

### Organ uptake of TG-derived FAs and glucose

Glycerol tri [^3^H]oleate ([^3^H]triolein [^3^H]TO; NET431L005MC, PerkinElmer)-labeled TG-rich lipoprotein (TRL)-like particles were prepared as previously described,^24^ and [^14^C]deoxy-d-glucose ([^14^C]DG; EC495A250UC, PerkinElmer) was added to the emulsion (5:1 ^3^H:^14^C activity ratio). Directly after the collection of 4-h fasted blood, mice (cohort 1) were intraperitoneally injected with 2 g kg^−1^ d-glucose to induce a standardized fed state while avoiding the production of endogenous GIP and GLP1 by the intestine. Half an hour later, mice were intravenously injected with the mixture of [^3^H]TO-labeled TRL-like particles (1 mg TG per mouse) and [^14^C]DG in 200 μL PBS, and killed by CO_2_ inhalation 15 min thereafter. After collecting blood via heart puncture to assess ALT activity (MAK052, Sigma–Aldrich), mice were perfused with ice-cold PBS. Collected tissues (max. 200 mg) were weighed and dissolved overnight at 55 °C in 500 μL Solvable (Perkin Elmer), after which 5 mL Ultima Gold liquid scintillation cocktail (PerkinElmer) was added to determine ^3^H and ^14^C activity using a Tri-Carb 2910 TR Low Activity Liquid Scintillation Analyzer (PerkinElmer). Uptake of [^3^H]TO- and [^14^C]DG-derived radioactivity by organs was expressed as the percentage of injected dose per gram of wet tissue.

### Liver histology

Parts of the liver (cohort 1 and 2) were fixed in formaldehyde, embedded in paraffin, cross-sectioned (5 μm) and stained for hematoxylin-eosin (H&E). In addition, fixed samples were dehydrated in 30% sucrose, cross-sectioned (5 μm) and stained with Oil red O. Liver lipid areas were quantified using ImageJ software (version 1.52a) on Oil red O-stained sections and expressed as the percentage of total tissue area. On H&E-stained sections, hepatocellular vesicular steatosis (*i.e.*, macrovesicular and microvesicular steatosis, separately) and hepatocellular hypertrophy were categorized 0–3, according to a NAFLD scoring system generated for rodent models.^25^

### Hepatic lipid contents

Liver lipids were extracted from snap-frozen liver samples (cohort 2) according to a modified protocol of Bligh and Dyer.^26^ Briefly, liver samples (approx. 50 mg) were homogenized in CH_3_OH (10 μL/mg tissue). To 45 μL homogenate, 1800 μL CH_3_OH:CHCl_3_ (1:3 v.v^−1^) was added. The organic phase obtained after centrifugation (20,000 g; 15 min; room temperature) was dried with a gentle flow of gas N_2_ and dissolved in 100 μL 2% Triton X-100 in CHCl_3_. After the second drying step, obtained samples were dissolved in 100 μL H_2_O for measurements. TGs and TC were measured as described above, and free FAs (NEFA-HR (2), Fujifilm) and protein (23225, Thermo Fisher Scientific) were measured using commercially available kits. Lipid contents were expressed as μmol/mg protein.

### Gene expression levels

Total RNA was extracted from snap-frozen liver samples (cohort 2) using TriPure RNA Isolation Reagent (11667165-001, Roche). After measuring RNA concentrations, 1 μg of RNA was reverse-transcribed into cDNA using Moloney Murine Leukemia Virus Reverse Transcriptase (M-MLV RT, Promega). Quantitative real-time PCR was performed using GoTaq® qPCR Master Mix (A6002, Promega) with a Bio-Rad CFX96 Touch™ Real-Time PCR Detection System. Expression levels were calculated as fold change relative to vehicle treatment using the 2^−ΔΔCT^ method and normalized to the mean of glyceraldehyde 3-phosphate dehydrogenase (*Gapdh*) and *Actb*. Primer sequences are listed in Supplementary Table S1.

### Fecal bile acid, energy and lipid excretion

Feces samples (cohort 2) were used to determine the feces excretion as well as fecal bile acid and energy excretion. Feces excretion was determined by weighing the samples after freeze-drying. Fecal bile acid excretion was calculated after measuring bile acid content in approx. 40–50 mg dried feces by liquid chromatography–mass spectrometry (LC/MS) as described before.^27^ Fecal energy excretion was derived from the caloric content of dried feces (approx. 300 mg) as measured by an oxygen bomb calorimeter (6100 Compensated Calorimeter, Parr Instrument Company). Fecal free FA and TC excretion was assessed as previously described.^28^

### Isolation of hepatic leukocytes

Fresh liver samples from cohort 2 were collected in ice-cold RPMI 1640 + Glutamax (61870-044, Thermo Fisher Scientific) from which hepatic leukocytes were isolated as described previously.^29^ In short, the tissues were minced and digested for 25 min at 37 °C in RPMI 1640 + Glutamax containing 1 mg/mL collagenase IV (C5138, Sigma–Aldrich), 1 mg/mL dispase II (D4693, Sigma–Aldrich), 2000 U/mL DNase I (D4263, Sigma–Aldrich) and 1 mg/mL collagenase D (11088866001, Roche). Digested liver samples were filtered through a 100 μm cell strainer and washed twice with PBS containing 0.5% bovine serum albumin (BSA) and 2 mM EDTA (PBS/BSA/EDTA), followed by centrifugation (300 *g*; 5 min; 4 °C). Obtained cell pellets containing leukocytes were treated with an erythrocyte lysis buffer (0.15 M NH_4_Cl, 1 mM KHCO_3_, and 0.1 mM Na_2_EDTA in H_2_O) for 2 min at room temperature. Remaining cells were washed with PBS/BSA/EDTA and pelleted once more as described above. After washing, total CD45^+^ leukocytes were isolated by means of magnetic-activated cell sorting (MACS) using LS columns and CD45 MicroBeads (130-052-301, Miltenyi Biotec) according to the manufacturer's protocol. After washing with PBS, obtained CD45^+^ leukocytes were pelleted and stained with Zombie NIR (423106, Biolegend) for 20 min at room temperature. After staining, the cells were washed with PBS and fixated with 1.9% paraformaldehyde for 15 min at room temperature. Fixated cells were washed with PBS/BSA/EDTA and further processed for flow cytometry.

### Flow cytometry

To analyze hepatic leukocyte subsets, isolated CD45^+^ leukocytes were incubated with a cocktail of antibodies directed against CD11c, CD19, Ly6G, F4/80, MHC-II, CLEC2, Siglec-F, CD64, NK1.1, CD11b, Ly6C, CD3, Thy1.2 and TIM4 (details regarding the antibodies are presented in Supplementary Table S2) in PBS/BSA/EDTA supplemented with True-Stain monocyte blocker (426103, Biolegend) and Brilliant Stain Buffer Plus (566385, BD biosciences) for 30 min at 4 °C. The stained samples were measured by spectral flow cytometry using a 3-laser Cytek Aurora spectral flow cytometer (Cytek Biosciences). Spectral unmixing was performed using SpectroFlo v3.0 (Cytek Biosciences). Gating of flow cytometry data was performed using FlowJoTM v10.8 Software (BD Biosciences). A representative gating strategy is presented in Supplementary Fig. S2A.

### Ethics statement

All mouse experiments were performed in accordance with the Institute for Laboratory Animal Research Guide for the Care and Use of Laboratory Animals and had received approval from the National Committee for Animal Experimentation of the Netherlands (“Centrale Commissie Dierproeven”; license number AVD1160020173305).

### Statistical analyses

Data are presented as mean ± SEM; mice and individual data points were only excluded in case of a technical reason. For all data, treatment effect was determined by two-way ANOVA and Tukey post-hoc analysis using Prism GraphPad version 9.3.1. Investigators were only blinded during the analyses of liver histology. *P* values less than 0.05 were considered statistically significant.

### Role of funders

Funders had no role in the study design, the collection, analyses and interpretation of data, the preparation of the manuscript, or decision to publish.

## Results

### Combined GIPR/GLP1R agonism lowers fat mass and food intake, accompanied by reduced plasma glucose and lipid levels

E3L.CETP mice were fed a HFHC run-in diet for 10 weeks. During the subsequent 10 weeks of intervention, vehicle-treated mice gained 5.3 ± 0.7 g of body weight and mice treated with the GIPR agonist gained 3.4 ± 0.5 g GLP1R agonism on the other hand completely mitigated body weight gain, and GIPR agonism combined with GLP1R agonism resulted in a 4.0 ± 0.7 g reduction in body weight (Fig. 1B). The differences in body weight gain between the intervention groups were explained by differences in fat mass (Fig. 1C) and not lean mass (Fig. 1D). Compared to vehicle-treated mice, concomitant GIPR/GLP1R agonism resulted in lower weight of the liver (−40%), gonadal white adipose tissue (gWAT; −63%), subcutaneous WAT (sWAT; −70%), interscapular brown adipose tissue (iBAT; −38%) and subscapular BAT (sBAT; −48%) (Fig. 1E and F). Throughout the intervention period, food intake was significantly reduced in the combined treatment group when compared to the vehicle group (−10%; Fig. 1G).

Fasting plasma levels of glucose, TGs and TC were monitored after 5 and 10 weeks of intervention, and were consistently decreased in mice treated with concomitant GIPR/GLP1R agonism when compared to vehicle-treated mice (Fig. 1H–J); two-way ANOVA suggests an additive effect of GIPR and GLP1R agonism in reducing plasma glucose after 5 weeks of intervention and in reducing plasma TG and TC after 10 weeks of intervention.

### Combined GIPR/GLP1R agonism improves glucose tolerance and stimulates the uptake of nutrients by BAT, WAT and the heart

**Fig. 2:**
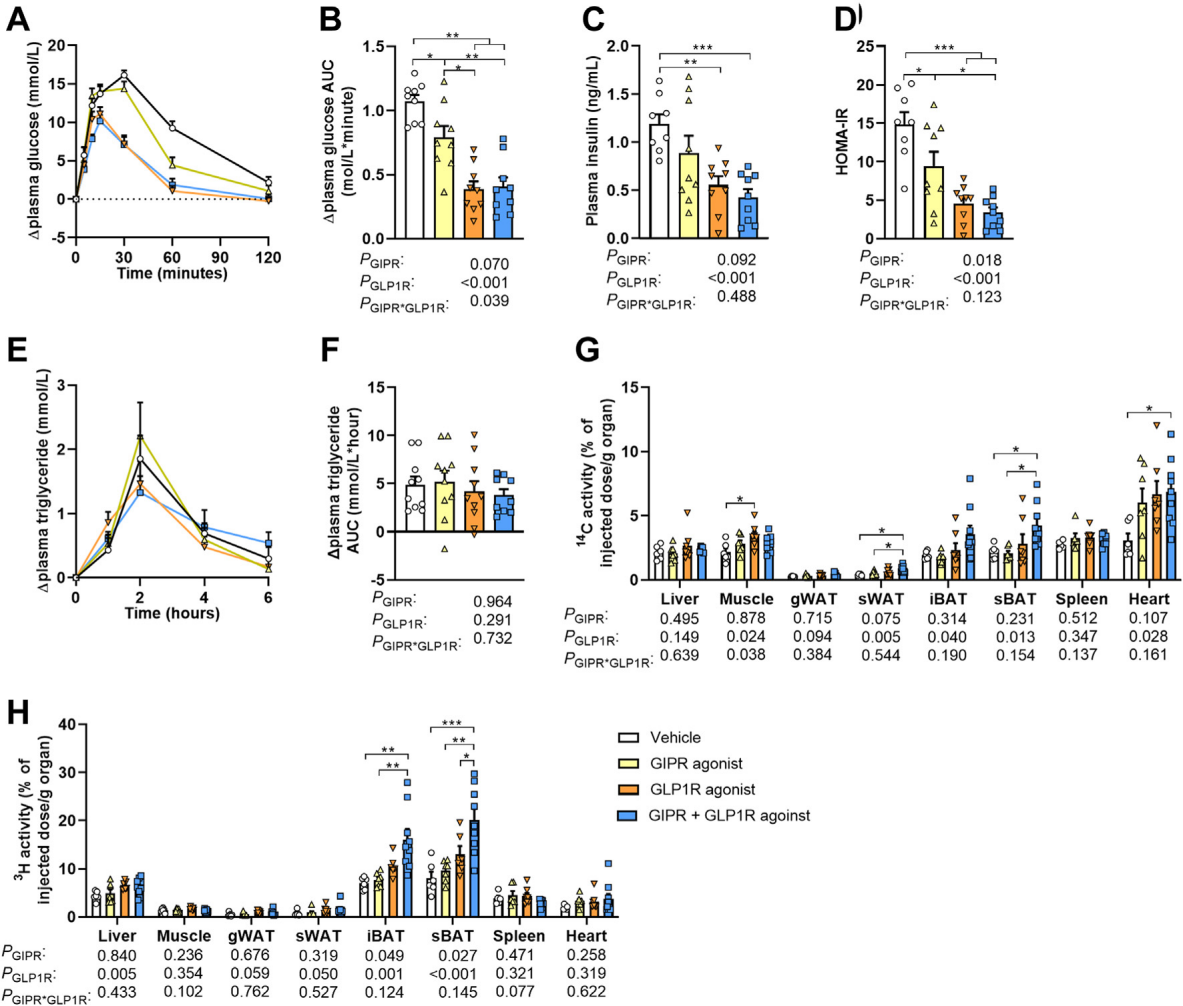
**Combined GIPR/GLP1R agonism improves glucose tolerance and stimulates the uptake of nutrients by brown adipose tissue, white adipose tissue and the heart.** Male APOE∗3-Leiden.CETP mice were fed a high-fat high-cholesterol diet and received subcutaneous injections with either a GIPR agonist (GIPFA-085; 300 nmol/kg), a GLP1R agonist (GLP-140; 30 nmol/kg), both agonists at these doses, or vehicle every other day. To determine oral glucose and lipid tolerance, mice received an oral administration of either d-glucose or olive oil after 5 weeks of treatment to determine plasma **(A)** glucose or **(E)** triglyceride excursion as change from baseline, from which **(B, F)**, the area under the curve (AUC) was determined. In plasma drawn at baseline in the oral glucose tolerance test, **(C)** insulin levels were also measured and used to calculate **(D)** the homeostatic model assessment for insulin resistance (HOMA-IR). After 10 weeks of treatment, the organ uptake of **(G)**^14^C-labeled deoxyglucose and **(H)** glycerol tri [^3^H]oleate-derived [^3^H]oleate from recombinant triglyceride-rich lipoprotein-like particles was determined. Data are presented as mean ± SEM and individual data points. **A**–**B***n* = 9 per group; **C**–**D***n* = 8–9 per group; **E**–**F***n* = 10 per group; **G**–**H***n* = 6–9. *P* values of two-way ANOVA are depicted below figure panels and symbols reflect statistical differences between groups as determined by Tukey post-hoc analysis with *∗P* < 0.05, *∗∗P* < 0.01 and *∗∗∗P* < 0.001. gWAT and sWAT, gonadal and subcutaneous white adipose tissue; iBAT and sBAT, interscapular and subscapular brown adipose tissue.

To investigate whether the reductions in plasma glucose and lipids upon combined GIPR/GLP1R agonism in the fasted state coincided with improvements in the postprandial state, an oral glucose and lipid tolerance test was performed after 5 weeks of treatment. Both GIPR agonism and GLP1R agonism improved oral glucose tolerance compared to vehicle treatment (AUC of −22% and −49% respectively; Fig. 2A and B). Combined agonism did not result in a further improvement, although the initial increase in plasma glucose levels seemed to be delayed in this group (Fig. 2A and B). GLP1R agonism also lowered fasting insulin levels when compared to vehicle treatment, while GIPR agonism only had no significant effect (Fig. 2C). Together with the reduction in fasting glucose levels (Fig. 1H), the HOMA-IR index was found to be lower for both GIPR and GLP1R agonism with no further improvement upon combined GIPR/GLP1R agonism (Fig. 2D). No statistically significant differences between the groups were observed in the overall oral lipid tolerance (Fig. 2E and F).

We next assessed the organ distribution of intravenously injected [^14^C]DG and [^3^H]TO-labeled TRL-like particles in a standardized fed state after 10 weeks of treatment. Single GIPR agonism did not affect the distribution of glucose and lipids, and single GLP1R agonism only increased the uptake of [^14^C]DG by muscle when compared to vehicle treatment. Combined GIPR/GLP1R agonism on the other hand significantly increased the uptake of [^14^C]DG by sWAT, sBAT and the heart, as primarily attributed to GLP1R agonism in the two-way ANOVA (Fig. 2G). Concomitant GIPR/GLP1R agonism additionally increased the uptake of [^3^H]TO-derived FAs by the iBAT and sBAT compared to vehicle, as explained by both GIPR and GLP1R agonism according to the two-way ANOVA (Fig. 2H).

Taken together, GLP1R agonism and combined GIPR/GLP1R agonism cause a pronounced improvement in glucose tolerance as (partly) explained by increased uptake of glucose by WAT and BAT. GIPR agonism also improves glucose tolerance, but we were unable to identify the contributing tissues, possibly due to the timing of experiments. Combined GIPR/GLP1R agonism furthermore increased the TG-derived FA-uptake by BAT as explained by both GIPR and GLP1R agonism, but while fasting TG levels were lower this did not translate into a significant improvement in oral lipid tolerance.

### Combined GIPR/GLP1R agonism attenuates hepatic steatosis

**Fig. 3:**
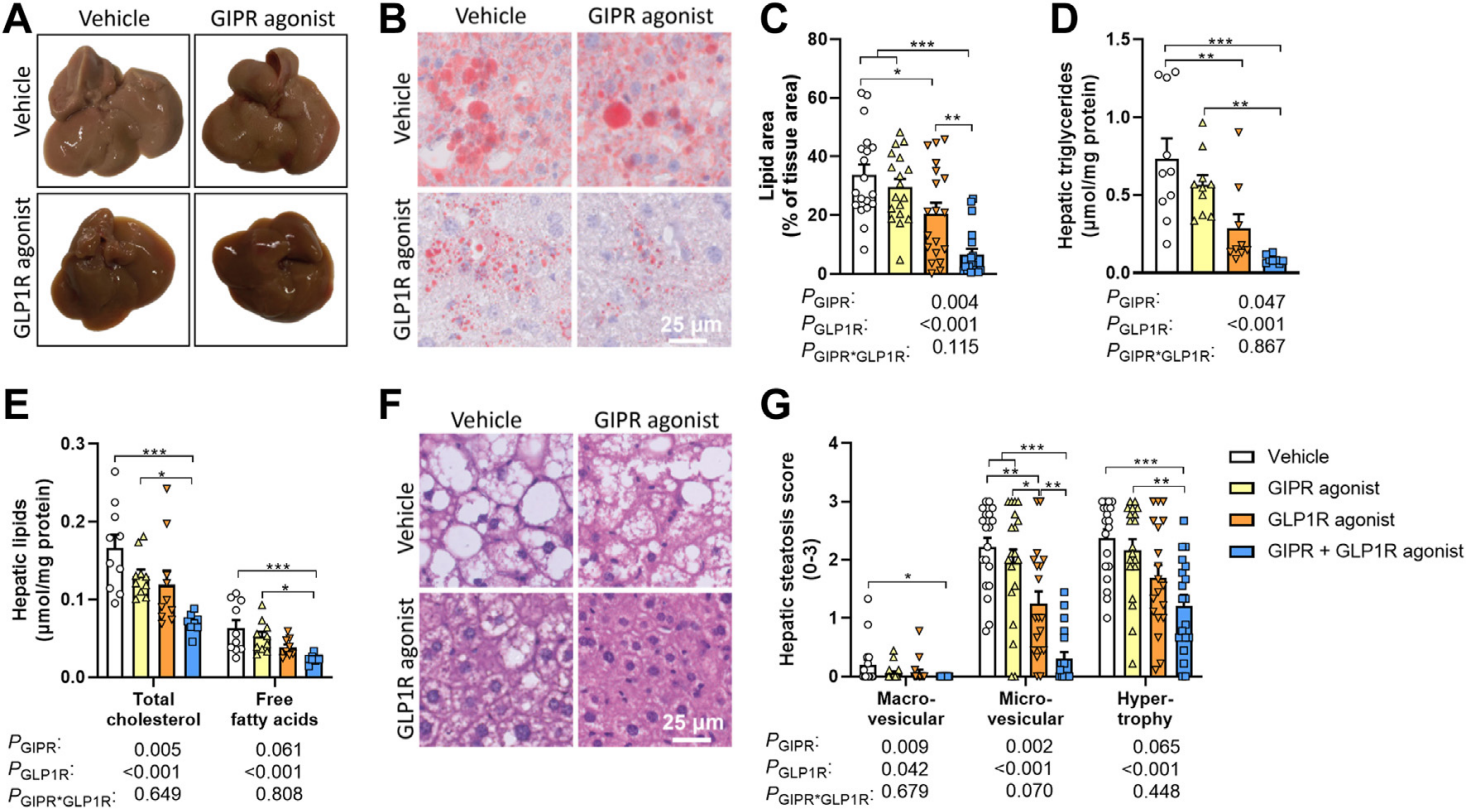
**Combined GIPR/GLP1R agonism lowers hepatic steatosis.** Male APOE∗3-Leiden.CETP mice were fed a high-fat high-cholesterol diet and received subcutaneous injections with either a GIPR agonist (GIPFA-085; 300 nmol/kg), a GLP1R agonist (GLP-140; 30 nmol/kg), both agonists at these doses, or vehicle every other day. After 10 weeks of treatment, **(A)** macroscopic pictures of representative livers were taken. **(B–C)** Cross-sections of the liver were stained with Oil red O to determine the hepatic lipid area. **(D**–**E)** Hepatic lipid content was assessed, and **(F**–**G)** NAFLD scores were determined on hematoxylin-eosin (H&E) stained cross-sections. Data are presented as mean ± SEM and individual data points. **C**, **G***n* = 18–19 per group; **D**–**E**, *n* = 8–10 per group. *P* values of two-way ANOVA are depicted below figure panels and symbols reflect statistical differences between groups as determined by Tukey post-hoc analysis with *∗P* < 0.05, *∗∗P* < 0.01 and *∗∗∗P* < 0.001.

To assess the effect of combined GIPR/GLP1R agonism on hepatic steatosis, livers were collected at the end of the 10-week intervention period. The livers from vehicle-treated mice exhibited an abnormal pale color as a feature of a fatty liver (Fig. 3A). In comparison, livers from mice treated with the GIPR agonist exhibited a slightly less pale appearance, while those of mice treated with the GLP1R agonist or both agonists combined showed a healthy, reddish color (Fig. 3A). Oil red O staining revealed that single GIPR agonism did not significantly lower hepatic lipid content as compared to vehicle treatment, while GLP1R agonism did (−39%; Fig. 3B and C). Strikingly, combining GIPR agonism with GLP1R agonism resulted in an additive reduction in hepatic lipid content (−80% *vs.* vehicle; Fig. 3B and C). Comparably, the hepatic TG content was lower upon GLP1R agonism, but not upon GIPR agonism when compared to vehicle treatment in the post-hoc analysis, while combining the two agonists additively reduced hepatic TG (−88%), TC (−58%) and free FA (−63%) content (Fig. 3D and E). In line with these effects, combined GIPR/GLP1R agonism strongly lowered the score for macrovascular steatosis as explained by both GIPR and GLP1R agonism in the two-way ANOVA (Fig. 3F and G). Furthermore, microvascular steatosis was lower upon GLP1R agonism, but not GIPR agonism, while combined GIPR/GLP1R agonism led to a pronounced reduction in microvascular steatosis even when compared to single GIPR and GLP1R agonism. Combined GIPR/GLP1R agonism, but not single agonism, correspondingly reduced hypertrophy scores (Fig. 3F and G).

### Combined GIPR/GLP1R agonism lowers the expression of lipogenic genes, and increases the expression of genes involved in cholesterol and bile acid synthesis in the liver

**Fig. 4:**
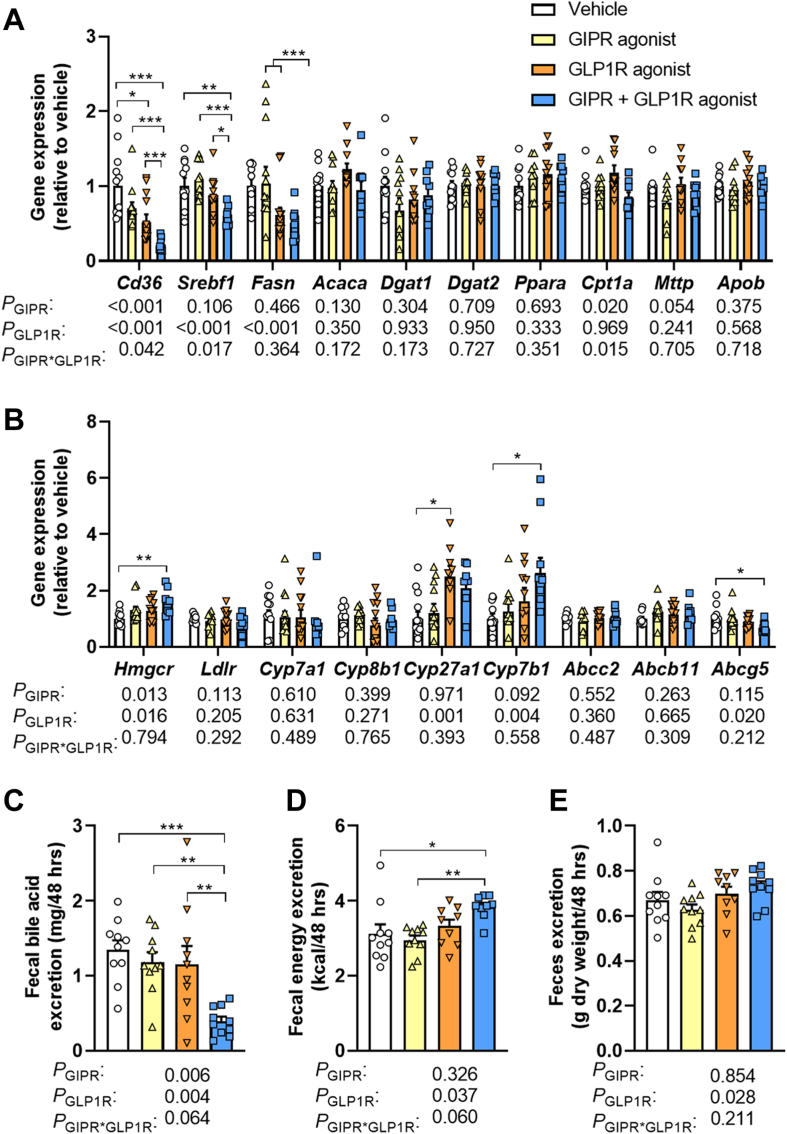
**Combined GIPR/GLP1R agonism lowers the expression of lipogenic genes, reduces fecal bile acid excretion and increases fecal energy excretion.** Male APOE∗3-Leiden.CETP mice were fed a high-fat high-cholesterol diet and received subcutaneous injections with either a GIPR agonist (GIPFA-085; 300 nmol/kg), a GLP1R agonist (GLP-140; 30 nmol/kg), both agonists at these doses, or vehicle every other day. After 10 weeks of treatment, relative mRNA expression levels of genes involved in **(A)** lipid metabolism and **(B)** cholesterol and bile acid metabolism were determined in the liver. Using feces samples collected in the eighth week of intervention, **(C)** fecal bile acid excretion, **(D)** fecal energy excretion, and **(E)** total fecal excretion were determined. Data are presented as mean ± SEM and individual data points. **A**–**B***n* = 8–10 per group; **C**–**D***n* = 9–10; **E***n* = 10. *P* values of two-way ANOVA are depicted below figure panels and symbols reflect statistical differences between groups as determined by Tukey post-hoc analysis with *∗P* < 0.05, *∗∗P* < 0.01 and *∗∗∗P* < 0.001. *Abcb11*, ATP binding cassette subfamily B member 11; *Abcc2*, ATP binding cassette subfamily C member 2; *Abcg5*, ATP binding cassette subfamily G member 5; *Acaca*, acetyl-CoA carboxylase α; *Apob*, apolipoprotein B; *Cd36*, cluster of differentiation 36; *Cpt1a*, carnitine palmitoyltransferase 1 α; *Cyp27a1*, cytochrome P450 family 27 subfamily A member 1; *Cyp7a1*, cytochrome P450 family 7 subfamily A member 1; *Cyp7b1*, cytochrome P450 family 7 subfamily B member 1; *Cyp8b1*, cytochrome P450 family 8 subfamily B member 1; *Dgat1*, diacylglycerol O-acyltransferase 1; *Dgat2*, diacylglycerol O-acyltransferase 2; *Fasn*, fatty acid synthase; *Hmgcr*, 3-hydroxy-3-methylglutaryl-CoA reductase; *Ldlr*, Low-density lipoprotein receptor; *Mttp*, Microsomal triglyceride transfer protein; *Ppara*, Peroxisome proliferator-activated receptor alpha; *Srebf1*, Sterol regulatory element-binding protein 1c.

To investigate additional mechanisms by which GIPR and GLP1R agonism might have contributed to the lowered hepatic lipid accumulation upon combined treatment, we first measured the hepatic expression of genes involved in lipid synthesis and secretion (Fig. 4A). Combined GIPR/GLP1R agonism lowered the expression of cluster of differentiation 36 (*Cd36*), important for the uptake of free FAs from the circulation (Fig. 4A). This was explained by a marked interaction between GIPR and GLP1R agonism. The combined treatment also reduced the expression of genes involved in *de novo* FA synthesis, including the transcription factor sterol regulatory element-binding protein 1 (*Srebf1*) also explained by an interaction of GIPR and GLP1R agonism, FA synthase (*Fasn*) as attributed to GLP1R agonism in the two-way ANOVA, but without affecting acetyl-CoA carboxylase (*Acaca*) expression (Fig. 4A). Expression of genes involved in the conversion of FAs to TGs (*i.e.,* diacylglycerol O-acyltransferase 1 and 2 (*Dgat1* and *Dgat2*)), FA oxidation (*i.e.,* peroxisome proliferator-activated receptor alpha (*Ppara*) and carnitine palmitoyltransferase 1α (*Cpt1a*)) or VLDL assembly (*i.e.,* microsomal triglyceride transfer protein (*Mttp*) and apolipoprotein B (*Apob*)) was not different between the groups (Fig. 4A).

With respect to cholesterol metabolism, we found no effect of the treatments on the expression of the low-density lipoprotein receptor (*Ldlr*) as a proxy for cholesterol uptake from the circulation (Fig. 4B). However, compared to vehicle combined GIPR/GLP1R agonism did increase the expression of 3-hydroxy-3-methylglutaryl-CoA reductase (*Hmgcr*), encoding the enzyme critical for *de novo* cholesterol synthesis, as explained by both GIPR and GLP1R agonism in the two-way ANOVA but with no significance for the single treatments in post-hoc analysis. There was no effect on the expression of genes involved in the classical pathway of bile acid synthesis (*i.e.,* cytochrome P450 family 7 subfamily A member 1 (*Cyp7a1*) and cytochrome P450 family 8 subfamily B member 1(*Cyp8b1*)) (Fig. 4B). In contrast, compared to vehicle single GLP1R agonism did increase the expression of cytochrome P450 family 27 subfamily A member 1 (*Cyp27a1*), and combined GIPR/GLP1R agonism increased the expression of cytochrome P450 family 7 subfamily B member 1 (*Cyp7b1*), both of which are involved in the alternative pathway of bile acid synthesis (Fig. 4B). Whilst expression of bile acid transporters (*i.e.,* ATP binding cassette subfamily C member 2 (*Abcc2*) and ATP binding cassette subfamily B member 11 (*Abcb11*)) was not affected by GIPR or GLP1R agonism, combined GIPR/GLP1R agonism did lower the expression of ATP binding cassette subfamily G member 5 (*Abcg5*), responsible for the biliary secretion of neutral sterols including cholesterol (Fig. 4B).

Taken together, combined GIPR/GLP1R agonism lowered the expression of genes involved in FA uptake, *de novo* FA synthesis, as well as sterol excretion in the liver, while increasing the expression of genes involved in *de novo* cholesterol synthesis and bile acid synthesis.

### Combined GIPR/GLP1R agonism reduces fecal bile acid excretion, coinciding with increased fecal energy excretion

**Supplementary Fig S1:**
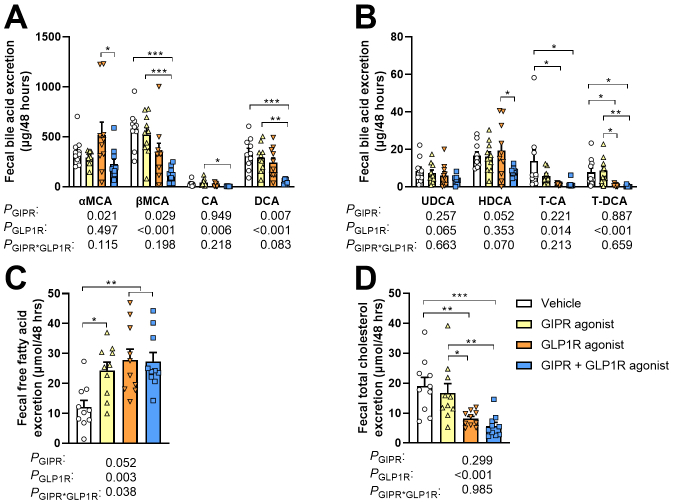


Bile acids are synthesized from cholesterol in the liver and are ultimately secreted into the small intestine to facilitate the emulsification and digestion of especially dietary lipids. The observed decrease in hepatic TC content and the increased expression of genes involved in bile acid synthesis may be indicative of an increased production and secretion of bile acids by the liver. Given that under steady-state conditions, the amount of bile acids excreted in feces is approximately equal to the production by the liver,^30^ we next measured the fecal bile acid excretion. Surprisingly, combined GIPR/GLP1R agonism strongly lowered the fecal excretion of both primary and secondary bile acids (Supplementary Fig. S1A and B), explained by the action of both GIPR and GLP1R agonism, leading to a 70% reduction in total fecal bile acid excretion as compared to vehicle treatment (Fig. 4C). This indicates a reduced rather than increased bile acid synthesis in the liver. To investigate whether the pronounced reduction in fecal bile acid excretion coincided with a hampered intestinal digestion and absorption of dietary energy substrates, we next assessed the fecal energy excretion. Indeed, combined GIPR/GLP1R agonism, but not the single treatments, increased the total fecal energy excretion as attributed to GLP1R agonism in the two-way ANOVA (Fig. 4D), with no changes in excretion of total feces mass (Fig. 4E). Accordingly, free FA excretion in the feces was increased upon combined GIPR/GLP1R agonism compared to vehicle, but also upon single treatments (Supplementary Fig. S1C). Combined GIPR/GLP1R agonism also decreased fecal cholesterol excretion compared to vehicle, as mainly attributed to GLP1R agonism (Supplementary Fig. S1D).

Taken together, the strongly lowered hepatic TC content upon combined GIPR/GLP1R agonism coincided with a pronounced decrease in fecal bile acid excretion and increased fecal energy excretion. These data suggest that the increased hepatic expression of *Hmgcr* and *Cyp7b1* and the reduced hepatic *Abcg5* expression as well as the decreased fecal cholesterol excretion are compensatory in an attempt to elevate the hepatic cholesterol content for bile acid synthesis. The question remains to what extent the reduced food intake, and which other mechanisms underly the strongly lowered circulating TC levels as well as the reduced hepatic TC content.

### Combined GIPR/GLP1R agonism reduces monocyte-derived Kupffer cells in the liver and lowers the hepatic expression of pro-inflammatory cytokines/chemokines

**Fig. 5:**
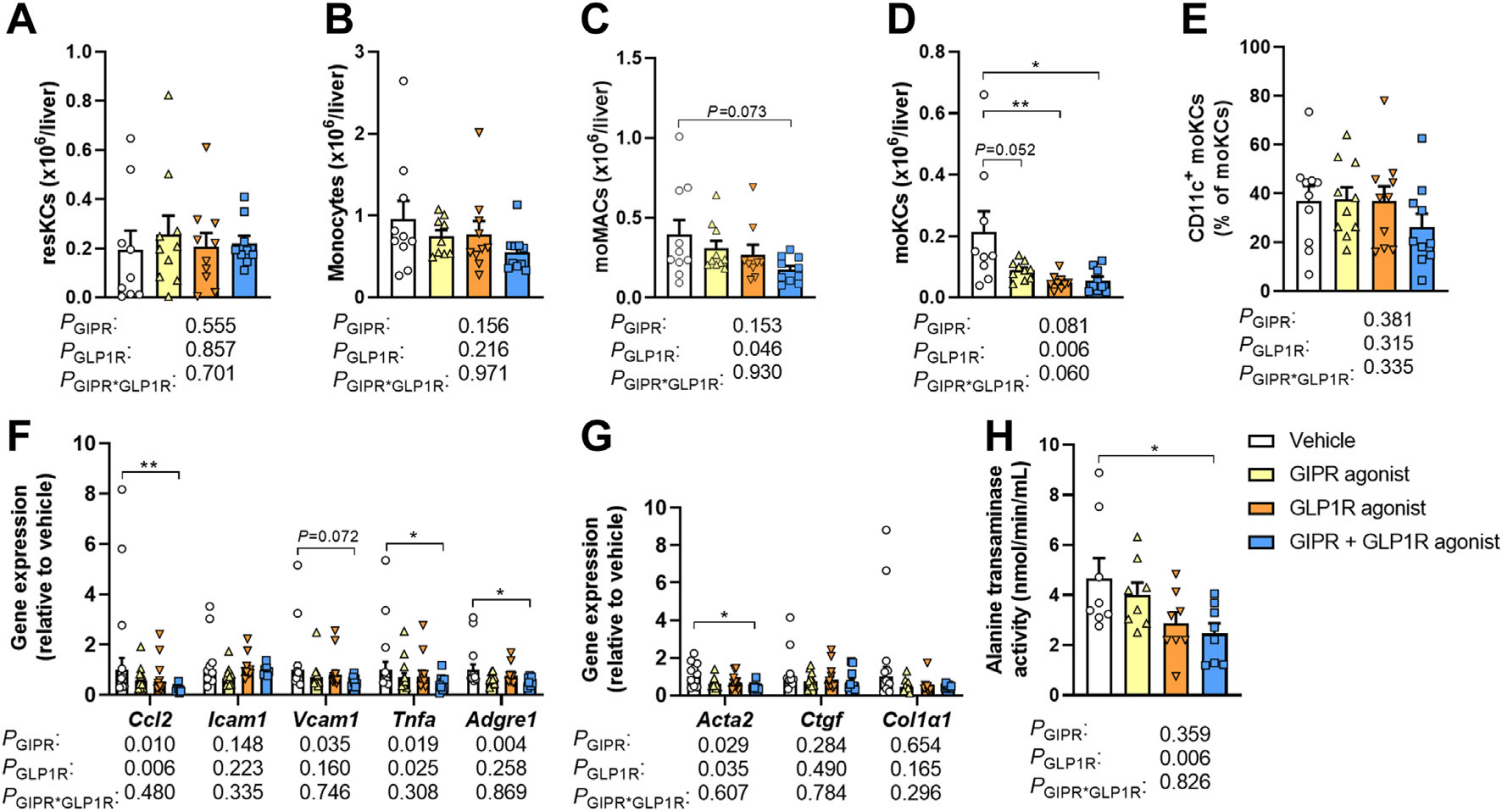
**Combined GIPR/GLP1R agonism reduces monocyte-derived Kupffer cells in the liver, lowers the hepatic expression of pro-inflammatory cytokines/chemokines and attenuates hepatic injury.** Male APOE∗3-Leiden.CETP mice were fed a high-fat high-cholesterol diet and received subcutaneous injections with either a GIPR agonist (GIPFA-085; 300 nmol/kg), a GLP1R agonist (GLP-140; 30 nmol/kg), both agonists at these doses, or vehicle every other day. After 10 weeks of treatment, livers were collected to quantify the number of **(A)** resident Kupffer cells (resKCs), **(B)** monocytes, **(C)** monocyte-derived macrophages (moMACs), **(D)** monocyte-derived Kupffer cells (moKCs) and **(E)** CD11c^+^ moKCs by flow cytometry. The hepatic mRNA expression of **(F)** cytokines/chemokines and macrophage markers was determined, as well as of **(G)** genes involved in hepatic injury. In plasma collected from heart puncture blood, **(H)** alanine transaminase activity was determined. Data are presented as mean ± SEM and individual data points. **A**–**E**, **H***n* = 9–10 per group; **F**–**G***n* = 8–10. *P* values of two-way ANOVA are depicted below figure panels and symbols reflect statistical differences between groups as determined by Tukey post-hoc analysis with *∗P* < 0.05, *∗∗P* < 0.01 and *∗∗∗P* < 0.001. *Acta2*, actin alpha 2; *Adgre1*, EGF-like module-containing mucin-like hormone receptor-like 1; *Casp3*, caspase 3; *Ccl2*, C–C motif chemokine ligand 2; *Col1a1*, collagen type 1α1; *Ctgf*, Connective tissue growth factor; *Icam1*, Intercellular adhesion molecule 1; *Tnfa*, Tumor necrosis factor α; *Vcam1*, Vascular cell adhesion protein 1.

**Supplementary Fig S2:**
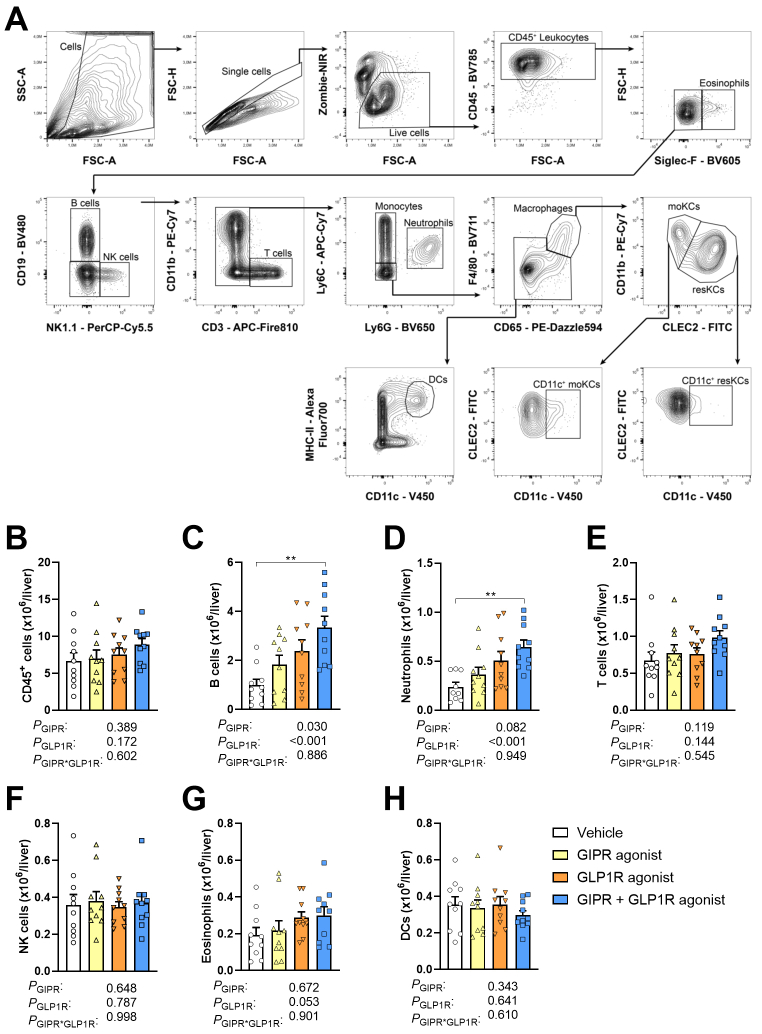


As NAFLD is driven by both hepatic steatosis and inflammation, we next evaluated hepatic inflammation by phenotyping immune cells in livers of 10 week-treated mice. GIPR and GLP1R agonism did not affect the total number of CD45^+^ cells (Supplementary Fig. S2B). Compared to vehicle, combined GIPR/GLP1R agonism increased the number of B cells and neutrophils in the liver (+235% and +171%, respectively; Supplementary Fig. S2C and D), as explained by both GIPR and GLP1R agonism in the two-way ANOVA with no significant effects of the single treatments in post-hoc analysis. GIPR and GLP1R agonism did not affect the number of T cells, natural killer (NK) cells, eosinophils or dendritic cells (DCs) (Supplementary Fig. S2E–H) and comparably did not affect the number of resident Kupffer cells (resKCs) or the number of monocytes in the liver (Fig. 5A and B). GLP1R agonism but not GIPR agonism reduced the number of monocyte-derived macrophages (moMACs) according to the two-way ANOVA (Fig. 5C). Strikingly, compared to vehicle, single GLP1R agonism significantly reduced the number of monocyte-derived KCs (moKCs), with no further decrease apparent upon combined GIPR/GLP1R agonism (Fig. 5D), while the percentage of the pro-fibrogenic^31^ CD11c^+^ subset of moKCs was not different between the treatment groups (Fig. 5E).

Possibly underlying these changes, combined GIPR/GLP1R agonism lowered the hepatic expression of C–C motif chemokine ligand 2 (*Ccl2*; −78% *vs.* vehicle) as attributed to both GIPR and GLP1R agonism in the two-way ANOVA, with no significant effect on the expression of vascular cell adhesion molecule 1 (*Vcam1*; −64% *vs.* vehicle), both of which are involved in the recruitment of circulating monocytes into the liver (Fig. 5F).^32^^,^^33^ In line with the reduction in moKCs, combined GIPR/GLP1R agonism furthermore lowered the hepatic expression of tumor necrosis factor *α* (*Tnfa;* −75%), mainly produced by macrophages, as explained by both agonists in the two-way ANOVA, as well as F4/80 (*Adgre1*; −48%), a widely used marker for murine macrophages (Fig. 5F).

### Combined GIPR/GLP1R agonism attenuates liver injury

Likely as a result of reduced steatosis and inflammation in the liver, the expression of α-smooth muscle actin (*Acta2*; −56%),^34^ which is expressed when resident hepatic stellate cells transform into myofibroblasts upon sensing liver injury and is involved in hepatic fibrogenesis, was lower in the livers of mice treated with combined GIPR/GLP1R agonism as compared to vehicle-treated mice (Fig. 5G). These effects were attributed to both GIPR and GLP1R agonism in two-way ANOVA, but neither the effect of single GIPR nor single GLP1R agonism reached statistical significance in the comparison with vehicle treatment in post-hoc analyses. The reduction in *Acta2* gene expression coincided with lowered plasma ALT activity upon combined GIPR/GLP1R agonism compared to vehicle (−47%; Fig. 5H) as explained by GLP1R agonism, which further supports the notion that the combined treatment attenuates liver injury.

## Discussion

Combined GIPR/GLP1R agonism is superior to single GLP1R agonism with regard to glycemic control and lowering body weight in patients with type 2 diabetes.^16^, ^17^, ^18^ In fact, the GIPR and GLP1R dual agonist tirzepatide (Mounjaro®) has recently been approved by FDA to treat type 2 diabetes in humans. However, the effect of combined GIPR/GLP1R agonism on NAFLD development is as of yet unknown. The current study in humanized E3L.CETP mice demonstrates that GIPR and GLP1R agonism additively attenuate hepatic steatosis, lower inflammation and ameliorate liver injury in the context of HFHC diet-induced NAFLD development.

In line with previous observations in mice^8^, ^9^, ^10^ and humans,^11^^,^^12^ we report that GLP1R agonism by itself attenuates hepatic steatosis, and now also show that the addition of GIPR agonism even further reduces lipid accumulation in the liver. Hepatic steatosis is triggered by excessive lipid storage in the liver, for which multiple underlying mechanisms have been suggested, including an overflow of nutrients towards the liver.^35^ In the current study GIPR and GLP1R agonism additively lowered food intake and body weight confirming previous reports in mice,^15^ which may at least in part explain the additive reduction of hepatic steatosis upon treatment with both agonists as it lowers the flux of nutrients towards the liver. The additive reduction in food intake may be mediated via the central nervous system, given that both single GIPR^14^^,^^36^ and GLP1R^37^^,^^38^ agonism have previously been shown to suppress food intake by activating the corresponding receptor on appetite-regulating neurons, and that the GIPR and GLP1R are expressed by the same as well as distinct neuronal populations.^13^

Besides lowering food intake, we observed that GIPR and GLP1R agonism exerted a synergistic effect on increasing fecal energy and lipid excretion, which likely contributed to the attenuated hepatic steatosis through lowering lipid availability. Previous reports in both hamsters and humans have shown that GLP1R agonism decreases intestinal lipid absorption and lowers the production of ApoB48-carrying lipoproteins,^39^, ^40^, ^41^, ^42^ as mediated both via peripheral and central GLP1R signaling.^39^, ^40^, ^41^ We now identified a decreased hepatic bile acid synthesis from cholesterol as an underlying mechanism given that bile acids facilitate the formation of mixed micelles to increase surface area for lipase activity in the intestinal lumen and allow for transport of hydrolyzed lipids to the intestinal brush border for subsequent absorption.^43^ An increased fecal lipid excretion was also observed upon apical sodium-dependent bile acid transporter (ASBT) inhibition.^44^^,^^45^ However, the mechanism of action differs from combined GIPR/GLP1R agonism as ASBT inhibition blocks the intestinal bile acid (re-)absorption. ASBT inhibition thus leads to a strongly increased, rather than decreased, fecal bile acid excretion; this effect is insufficiently compensated for by the liver, thereby hampering lipid absorption by the intestine. Since cholesterol is the main precursor of bile acids, the lowered hepatic bile acid synthesis may be related to the reduction in hepatic cholesterol content that we observed upon combined GIPR/GLP1R agonism, which in itself may at least in part be explained by the lowered dietary intake of the cholesterol-rich diet. Interestingly, combined GIPR/GLP1R agonism increased the hepatic expression of genes involved in bile acid synthesis, while increasing expression of the rate-limiting enzyme in *de novo* cholesterol synthesis (*Hmgcr*) and lowering the expression of *Abcg5* involved in sterol excretion, which was in parallel with reduced fecal cholesterol excretion. Collectively, these findings indicate a compensatory response attempting to restore hepatic cholesterol content for bile acid synthesis in line with our previous observations for single GLP1R agonism.^10^

Another mechanism potentially contributing to less lipid accumulation in the liver was the increased uptake of postprandial glucose and TRL-TG-derived FAs by extrahepatic tissues upon combined GIPR/GLP1R agonism. Previous studies in mice have reported that GLP1R agonism with exendin-4 increased the uptake of glucose and TG-derived FAs by BAT and WAT via activating BAT and inducing browning of WAT, respectively, as mediated via increased sympathetic outflow towards the tissues.^46^ GIPR agonism may have added to this by facilitating the nutrient flow to and lipid hydrolysis in the adipose tissue in the postprandial state, given that GIP infusion in humans has previously been shown to increase blood flow in abdominal Swat^47^ and GIPR agonism in human adipocytes has furthermore been shown to increase both the expression and activity of lipoprotein lipase,^48^^,^^49^ which is crucial for the liberation and subsequent uptake of FAs from TRL-TGs. Consequently, GIPR agonism may thereby prevent the spill-over of postprandial lipids towards the liver. Given that GIPR agonism has also been reported to stimulate intracellular lipolysis in WAT in the fasted state,^17^ it may induce energy wasting due to lipid cycling as well.

In addition to attenuating hepatic steatosis, combined GIPR/GLP1R agonism strongly lowered hepatic inflammation as evidenced by a decreased number of moKCs possibly as a result of lowered expression of hepatic chemokines involved in leukocyte trafficking to the liver. Here, the combined treatment did not outperform single GLP1R agonism despite that both treatments reduced hepatic moKCs, which may indicate that maximal effect on inflammation was already achieved by single GLP1R agonism at the used dose. Our findings are in line with previous studies showing that GLP1R agonism using exendin-4 reduces macrophage recruitment into the liver in mice, coinciding with lowered expression of *Ccl2*,^50^ and that in patients with NASH the GLP1R agonist liraglutide reduces the circulating levels of monocyte chemoattractant protein 1 (MCP-1).^51^ With a comparable mechanism, overexpression of GIP has also been shown to lower macrophage infiltration into the vessel wall in mice.^52^ The anti-inflammatory effects of GIPR and GLP1R agonism may be indirect and a consequence of attenuated hepatic steatosis, as lipid accumulation in itself is a potent inducer of inflammation.^53^ However, given that both the GIPR^19^ and the GLP1R^54^ are also expressed by at least a proportion of immune cells including monocytes and macrophages, GIPR and GLP1R agonism may also exert anti-inflammatory effects via direct engagement with immune cells, for example by lowering *Ccl2* expression in macrophages as has been shown for GLP1R agonism.^55^

In conclusion, combined GIPR/GLP1R agonism additively attenuates hepatic steatosis and lowers hepatic inflammation, ameliorating liver injury during the development of NAFLD in E3L.CETP mice. Given that this mouse model is a well-established model for human insulin resistance, diabetic dyslipidemia and NAFLD, and that concomitant GIPR/GLP1R agonism with tirzepatide was shown to lower NASH-related blood biomarkers in patients with type 2 diabetes,^56^ we anticipate that combined GIPR/GLP1R agonism is a promising strategy for the prevention/treatment of NAFLD in humans. This hypothesis is currently tested with the SYNERGY-NASH phase 2 clinical trial, which assesses the efficacy of tirzepatide to prevent the worsening of fibrosis, decrease NAFLD activity scores and decrease the liver fat content in patients with NASH.

## Contributors

Conceptualization, Z.Y., R.V.E., P.C.N.R. and S.K.; Formal Analysis, Z.Y. and R.V.E.; Data verification, Z.Y. and R.V.E.; Investigation, Z.Y., R.V.E., X.G., C.V.M, J.M.L., B.G., M.G., J.F.D.B, Y.W. and S.K.; Writing—Original Draft, Z.Y.; Writing—Review & Editing, Z.Y., R.V.E., T.C., H.Q., M.R.B., P.C.N.R. and S.K.; Supervision, S.K.; Funding acquisition, P.C.N.R and S.K. Both Z.Y. and R.V.E. contributed equally. All authors have read and approved the final version of the manuscript.

## Data sharing statement

The datasets used and/or analyzed during the current study are available from the corresponding author on reasonable request.

## Declaration of interests

HQ and TC are employees and shareholders of Eli Lilly and Company. Eli Lilly and Company had no role in study design, data collection and analysis or decision to publish.

## Figures and Tables

**Graphical abstract:**