# Supplementary material for: Combined GIP receptor and GLP1 receptor agonism attenuates NAFLD in male APOE∗3-Leiden.CETP mice
Source: eBioMedicine. 2023 Jun 26;93:104684. doi: 10.1016/j.ebiom.2023.104684 (PMC10318458; doi:10.1016/j.ebiom.2023.104684)
Supplement: Supplementary Figs S1–S2 and Tables S1–S2 [file mmc1.docx]

Combined GIP receptor and GLP1 receptor agonism attenuates NAFLD in male APOE*3-Leiden.CETP mice

Zhixiong Ying*^1,2^, Robin van Eenige*^1,2^, Xiaoke Ge^1,2^, Christy van Marwijk^1,2^, Joost M. Lambooij^3,4^, Bruno Guigas^3^, Martin Giera^5^, Jan Freark de Boer^6^, Tamer Coskun^7^, Hongchang Qu^7^, Yanan Wang^1,8^, Mariëtte R. Boon^1,2^, Patrick C.N. Rensen^1,2^, Sander Kooijman^#1,2^

^*^Authors contributed equally.

^1^Division of Endocrinology, Department of Medicine, Leiden University Medical Center, Leiden, The Netherlands

^2^Einthoven Laboratory for Experimental Vascular Medicine, Leiden University Medical Center, Leiden, The Netherlands

^3^Department of Parasitology, Leiden University Medical Center, Leiden, The Netherlands

^4^Department of Cell and Chemical Biology, Leiden University Medical Center, Leiden, The Netherlands

^5^The Center for Proteomics and Metabolomics, Leiden University Medical Center, Leiden, The Netherlands

^6^Departments of Pediatrics and Laboratory Medicine, University of Groningen, University Medical Center Groningen, Groningen, The Netherlands

^7^Department of Diabetes/Endocrine, Lilly Research Laboratories, Lilly Corporate Center, Indianapolis, IN, United States

^8^Med-X institute, Center for Immunological and Metabolic Diseases and Department of Endocrinology, First Affiliated Hospital of Xi'an Jiaotong University, Xi'an Jiaotong University, Xi’an, China.

**Table S1. Primer sequences for quantitative real-time PCR.**

|  | Primers | |
| --- | --- | --- |
| Gene | Forward | Reverse |
| *Abcb11* | CTGCCAAGGATGCTAATGCA | CGATGGCTACCCTTTGCTTCT |
| *Abcc2* | GCTTCCCATGGTGATCTCTTC | ATCATCGCTTCCCAGGTACTG |
| *Abcg5* | GAGCTGCAGAGGATGATTGCT | AGCCACCCTGGTCTTGGA |
| *Acaca* | AACGTGCAATCCGATTTGTT | GAGCAGTTCTGGGAGTTTCG |
| *Acta2* | CCTGACGGGCAGGTGATC | ATGAAAGATGGCTGGAAGAGAGTCT |
| *Actb* | AACCGTGAAAAGATGACCCAGAT | CACAGCCTGGATGGCTACGTA |
| *Adgre1* | CTTTGGCTATGGGCTTCCAGTC | GCAAGGAGGACAGAGTTTATCGTG |
| *Apob* | GCCCATTGTGGACAAGTTGATC | CCAGGACTTGGAGGTCTTGGA |
| *Ccl2* | GCATCTGCCCTAAGGTCTTCA | TTCACTGTCACACTGGTCACTCCTA |
| *Cd36* | GCAAAGAACAGCAGCAAAATC | CAGTGAAGGCTCAAAGATGG |
| *Col1a1* | GAGAGAGCATGACCGATGGATT | TGTAGGCTACGCTGTTCTTGCA |
| *Cpt1a* | GAGACTTCCAACGCATGACA | ATGGGTTGGGGTGATGTAGA |
| *Ctgf* | GGCCTCTTCTGCGATTTCG | CCATCTTTGGCAGTGCACACT |
| *Cyp27a1* | TCTGGCTACCTGCACTTCCT | CTGGATCTCTGGGCTCTTTG |
| *Cyp7a1* | CAGGGAGATGCTCTGTGTTCA | AGGCATACATCCCTTCCGTGA |
| *Cyp7b1* | CAGCTATGTTCTGGGCAATG | TCGGATGATGCTGGAGTATG |
| *Cyp8b1* | GGACAGCCTATCCTTGGTGA | CGGAACTTCCTGAACAGCTC |
| *Dgat1* | TCCGTCCAGGGTGGTAGTG | TGAACAAAGAATCTTGCAGACGA |
| *Dgat2* | TCGCGAGTACCTGATGTCTG | CTTCAGGGTGACTGCGTTCT |
| *Fasn* | GCGCTCCTCGCTTGTCGTCT | TAGAGCCCAGCCTTCCATCTCCTG |
| *Gapdh* | GGGGCTGGCATTGCTCTCAA | TTGCTCAGTGTCCTTGCTGGGG |
| *Hmgcr* | CCGGCAACAACAAGATCTGTG | ATGTACAGGATGGCGATGCA |
| *Icam1* | TCCGCTGTGCTTTGAGAAC | TCCGGAAACGAATACACGG |
| *Ldlr* | GCATCAGCTTGGACAAGGTGT | GGGAACAGCCACCATTGTTG |
| *Mttp* | CTCTTGGCAGTGCTTTTTCTCT | GAGCTTGTATAGCCGCTCATT |
| *Ppara* | ATGCCAGTACTGCCGTTTTC | GGCCTTGACCTTGTTCATGT |
| *Srebf1* | AGCCGTGGTGAGAAGCGCAC | ACACCAGGTCCTTCAGTGATTTGCT |
| *Tnfa* | AGCCCACGTCGTAGCAAACCAC | TCGGGGCAGCCTTGTCCCTT |
| *Vcam1* | TGGAGGTCTACTCATTCCC | GACAGGTCTCCCATGCACA |

*Abcb11*, ATP binding cassette subfamily B member 11; *Abcc2*, ATP binding cassette subfamily C member 2; *Abcg5*, ATP binding cassette subfamily G member 5*; Acaca*, acetyl-CoA carboxylase α; *Acta2*, actin alpha 2; *Actb*, β-actin; *Adgre1*, EGF-like module-containing mucin-like hormone receptor-like 1; *Apob*, apolipoprotein B; *Ccl2*, C-C motif chemokine ligand 2; *Cd36*, cluster of differentiation 36; *Col1a1*, collagen type 1α1; *Cpt1a*, carnitine palmitoyltransferase 1 α; *Ctgf*, connective tissue growth factor; *Cyp27a1*, cytochrome P450 family 27 subfamily A member 1 ; *Cyp7a1*, cytochrome P450 family 7 subfamily A member 1; *Cyp7b1*, cytochrome P450 family 7 subfamily B member 1; *Cyp8b1*, cytochrome P450 family 8 subfamily B member 1; *Dgat1*, diacylglycerol O-acyltransferase 1; *Dgat2*, diacylglycerol O-acyltransferase 2; *Fasn*, fatty acid synthase; *Gapdh*, glyceraldehyde 3-phosphate dehydrogenase; *Glut1*, glucose transporter 1; *Glut4*, glucose transporter 4; *Hmgcr*, 3-hydroxy-3-methylglutaryl-CoA reductase; *Icam1*, intercellular adhesion molecule 1; *Ldlr*, low-density lipoprotein receptor; *Mttp*, microsomal triglyceride transfer protein; *Ppara*, peroxisome proliferator activated receptor α; *Srebf1*, sterol regulatory element-binding protein 1c; *Tnfa*, tumor necrosis factor α; *Vcam1*, vascular cell adhesion protein 1.

**Table S2. Antibodies used for flow cytometry.**

| **Target** | **Clone** | **Conjugate** | **Source** | **Catalog number** | **RRID** |
| --- | --- | --- | --- | --- | --- |
| CD3 | 17A2 | APC/Fire-810 | Biolegend | 100267 | AB_2876392 |
| CD11b | M1/70 | PE-Cy7 | eBioscience | 25-0112-82 | AB_469588 |
| CD11c | HL3 | V450 | BD Biosciences | 560521 | AB_1727423 |
| CD19 | 1D3 | BV480 | BD Biosciences | 566107 | AB_2739509 |
| CD45 | 30-F11 | BV785 | Biolegend | 103149 | AB_2564590 |
| CD64 | X54-5/7.1 | PE-DAZZLE594 | Biolegend | 139320 | AB_2566559 |
| CD90.2 | 30-H12 | Alexa Fluor 700 | Biolegend | 105319 | AB_493724 |
| CLEC2 | 17D9 | FITC | Bio-Rad | MCA5700 | AB_11152776 |
| F4/80 | BM8 | BV711 | Biolegend | 123147 | AB_2564588 |
| Ly6C | HK1.4 | APC-Cy7 | Biolegend | 128025 | AB_10643867 |
| Ly6G | 1A8 | BV650 | Biolegend | 127641 | AB_2565881 |
| MHC-II | M5/114.15.2 | BV750 | BD Biosciences | 747458 | AB_2872135 |
| MHC-II | M5/114.15.2 | Alexa Fluor 700 | Thermo Fisher | 56-5321-82 | AB_494009 |
| NK1.1 | PK136 | PerCP-Cy5.5 | Biolegend | 108727 | AB_2132706 |
| Siglec-F | E50-2440 | PE | BD Biosciences | 552126 | AB_394341 |
| Siglec-F | E50-2440 | BV605 | BD Biosciences | 740388 | AB_2740118 |
| TIM4 | 54 (RMT4-54) | PerCP-eFluor710 | Thermo Fisher | 46-5866-82 | AB_2573781 |


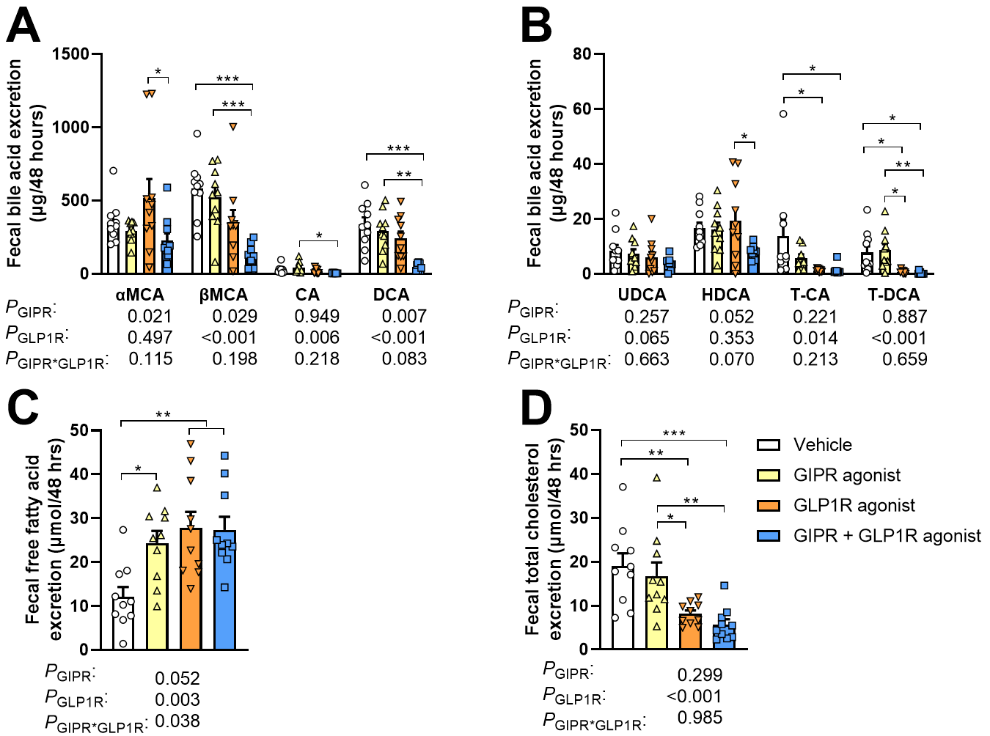


**Figure S1. Combined GIPR/GLP1R agonism reduces fecal bile acid and cholesterol excretion and increases fecal free fatty acid excretion.** Male APOE*3-Leiden.CETP mice were fed a high-fat high-cholesterol diet and received subcutaneous injections with either a GIPR agonist (GIPFA-085; 300 nmol/kg), a GLP1R agonist (GLP-140; 30 nmol/kg), both agonists at these doses, or vehicle every other day. After the 8^th^ week of treatment, feces samples were collected to measure fecal **(A-B)** bile acid, **(C)** free fatty acid and **(D)** total cholesterol excretion. **A-B** *n*=8-10 per group; **C-D** *n*=10 per group. *P* values of two-way ANOVA are depicted below figure panels and symbols reflect statistical differences between groups as determined by Tukey post-hoc analysis with **P*<0.05, ***P*<0.01 and ****P*<0.001. CA, cholic acid; DCA, deoxycholic acid; HDCA, hyodeoxycholic acid; T-CA, taurocholic acid; T-DCA, taurodeoxycholic acid; UDCA, ursodeoxycholic acid; αMCA, α-muricholic acid; βMCA, β-muricholic acid.


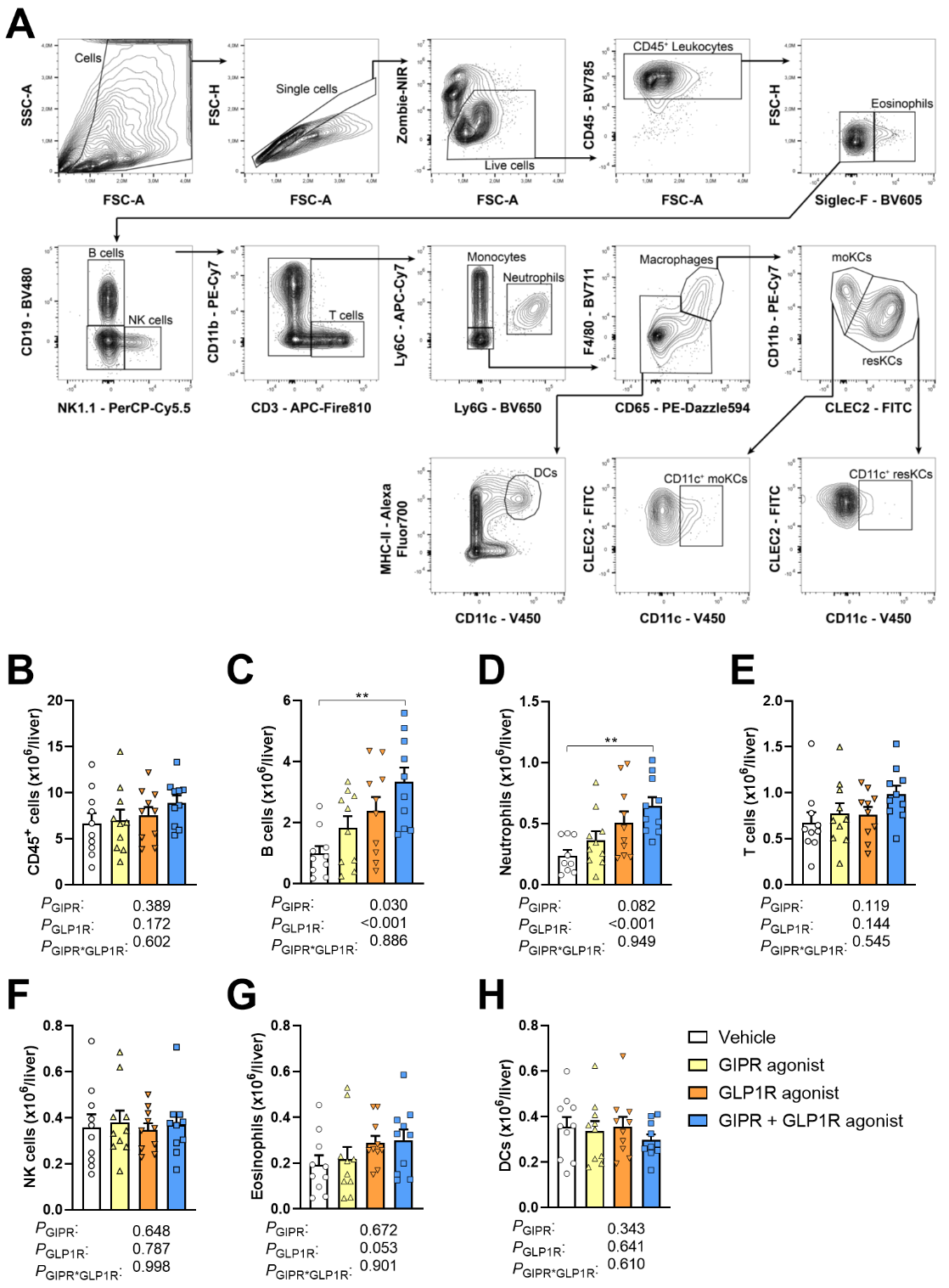


**Figure S2. Combined GIPR/GLP1R agonism increases B cells and neutrophils in the liver**. Male APOE*3-Leiden.CETP mice were fed a high-fat high-cholesterol diet and received subcutaneous injections with either a GIPR agonist (GIPFA-085; 300 nmol/kg), a GLP1R agonist (GLP-140; 30 nmol/kg), both agonists at these doses, or vehicle every other day. After 10 weeks of treatment, livers were collected for flow cytometry. **(A)** A representative gating strategy is presented. Next, the number of **(B)** CD45^+^ cells, **(C)** B cells, **(D)** neutrophils, **(E)** T cells, **(F)** natural killer (NK) cells, **(G)** eosinophils and **(H)** dendritic cell (DCs) were quantified. Data are presented as mean ± SEM and individual data points. **B**-**C**, **E**-**H** *n*=10 per group; **D** n=9-10. *P* values of two-way ANOVA are depicted below figure panels and symbols reflect statistical differences between groups as determined by Tukey post-hoc analysis with **P*<0.05, ***P*<0.01 and ****P*<0.001.
